# Associations between non-motor symptoms and patient characteristics in Parkinson’s disease: a multicenter cross-sectional study

**DOI:** 10.3389/fnagi.2023.1252596

**Published:** 2023-09-06

**Authors:** Remi Morimoto, Mutsumi Iijima, Yasuyuki Okuma, Keisuke Suzuki, Fumihito Yoshii, Shigeru Nogawa, Takashi Osada, Kazuo Kitagawa

**Affiliations:** ^1^Department of Neurology, School of Medicine, Tokyo Women's Medical University, Tokyo, Japan; ^2^Department of Neurology, Juntendo University Shizuoka Hospital, Shizuoka, Japan; ^3^Department of Neurology, Dokkyo Medical University, Tochigi, Japan; ^4^Department of Neurology, Saiseikai Shonan Hiratsuka Hospital, Kanagawa, Japan; ^5^Department of Neurology, Tokai University Hachioji Hospital, Tokyo, Japan; ^6^Department of Neurology, National Center of Neurology and Psychiatry, Tokyo, Japan

**Keywords:** Parkinson’s disease, non-motor symptoms, older adults, self-questionnaire, Japan

## Abstract

**Objective:**

Parkinson’s disease (PD) is characterized by various non-motor symptoms (NMS), such as constipation, olfactory disturbance, sleep disturbance, mental disorders, and motor symptoms. This study aimed to investigate factors associated with NMS in patients with PD.

**Methods:**

Symptoms of PD were evaluated using the Movement Disorder Society Unified Parkinson’s Disease Rating Scale (MDS-UPDRS), Parts I–IV. NMS was assessed using the MDS-UPDRS Part I (self-assessment of NMS) and rapid eye movement sleep behavior disorder (RBD) questionnaires. Patients were categorized by age into <70 years and ≥ 70 years (older adults) groups, according to disease duration into early-stage and advanced-stage groups with a cut-off value of 5 years for motor symptoms, and by sex into male and female groups.

**Results:**

A total of 431 patients with PD (202 males and 229 females) with a mean age of 67.7 years, a mean disease duration of 6.4 years, and a mean Part I total score of 9.9 participated in this study. The Part I total score was significantly positively correlated (*p* < 0.01) with disease duration and Part II, III, and IV scores. For Part I sub-item scores, the older group had significantly higher scores for cognitive impairment, hallucinations, sleep problems, urinary problems, and constipation than the <70 years group, whereas the advanced-stage group had significantly higher scores for hallucinations, sleep problems, daytime sleepiness, pain, urinary problems, and constipation (*p* < 0.05) than the early-stage group. Anxiety was higher in female patients than in male patients, whereas daytime sleepiness, urinary problems, and RBD were higher in male patients than in female patients (*p* < 0.05). Factors affecting Part I included disease duration, Part II total scores, Part IV total scores, and RBD.

**Conclusion:**

According to the self-questionnaire assessment, NMS was highly severe in older adult patients, those with longer illness duration, subjective and objective motor function impairments, and RBD. Sex-based differences were also observed.

## 1. Introduction

Parkinson’s disease (PD) is a neurodegenerative disease that causes various non-motor symptoms (NMS), including constipation, olfactory disturbances, sleep disturbances, psychiatric disorders, and motor symptoms (Marinus et al., 2018). Autonomic neuropathy symptoms, including constipation and frequent urination, along with NMS, such as rapid eye movement sleep behavior disorder (RBD), olfactory dysfunction, and depression, are frequently observed before the onset of motor symptoms. Insomnia, neurogenic bladder, and cognitive impairment develop after the onset of motor symptoms, with the progression of the PD (Savica et al., 2010; Kalia and Lang, 2015). Olfactory disturbances and RBD in NMS are considered clinical markers for the early diagnosis of PD because they are highly disease-specific and appear earlier than motor symptoms (Jellinger, 2011; Lang, 2011; Martinez-Martin et al., 2011).

NMS and motor symptoms are associated with health-related quality of life (HRQOL) (Zhao et al., 2021). In contrast, NMS have been reported to affect HRQOL more than motor symptoms, and its progression contributes to reduction in HRQOL (Martinez-Martin et al., 2011). In Japan, an association between the NMS and HRQOL has been reported in patients with advanced-stage PD (Maeda et al., 2017). However, 31.8–65.2% of patients PD experiencing NMS do not report these disturbances to their physicians; therefore, they may remain untreated (Chaudhuri et al., 2010). Capturing the characteristics of patients’ NMS and practicing personalized medicine to improve HRQOL are important for clinicians.

Additionally, there are approximately 2.9 million patients with PD in Japan, among them, 85% are aged 65 years or older, and this number is expected to increase with an aging population (Mori and Abe, 2017). Regarding the association between older patients with PD and quality of life (QOL), QOL in patients with middle-aged-onset PD (50–69 years) was associated with the Movement Disorder Society-sponsored revision of the Unified Parkinson’s Disease Rating Scale (MDS-UPDRS) Part III and NMS, whereas QOL in patients with old-onset PD (≥70 years) was associated with NMS (Park et al., 2018). To improve the HRQOL of patients with PD, evaluating NMS and examining its associated factors may be crucial. The MDS-UPDRS Part I is a comprehensive framework for identifying NMS and was revised in 2007 based on a growing understanding of the impact of NMS on patients’ lives (Goetz et al., 2007). This study aimed to assess the characteristics of NMS in PD and the differences in NMS based on age, sex, and disease duration, and to examine the factors involved in NMS using the MDS-UPDRS Part I.

## 2. Methods

### 2.1. Participants

This study included ambulatory patients with PD who had achieved a Mini-Mental State Examination (MMSE) score of 24 or higher, who were registered between February 1, 2015 and July 31, 2022. The participating institutions were the Tokyo Women’s Medical University Hospital, Juntendo University Shizuoka Hospital, Dokkyo Medical University Hospital, Tokai University Hospital, Tokai University Hachioji Hospital, and Keio University Hospital. The clinical diagnosis of PD was based on UK Brain Bank Criteria (Hughes et al., 1992). The exclusion criteria included individuals with dementia or those who scored < 24 on the MMSE.

### 2.2. Evaluation of clinical symptoms

Age, sex, and disease duration were also recorded. PD severity was assessed using the Hoehn and Yahr (HY) scale, and clinical symptoms were assessed using the Japanese version of the MDS-UPDRS, Parts I–IV (Kashihara et al., 2014). HY scale was evaluated in the “on” patient status. Part I of the MDS-UPDRS is a self-assessment of NMS in daily life, in which the patient or caregiver is asked to complete a 13-item questionnaire (cognitive impairment, hallucinations, depressed mood, anxious mood, apathy, dopamine dysregulation syndrome (DDS), sleep problems, daytime sleepiness, pain, urinary problems, constipation, lightheadedness in standing, and fatigue); Part II is a self-assessment of motor symptoms in daily life, in which the patient or caregiver must complete a 13-item questionnaire; Part III is an investigation of the presence of 18 possible motor symptoms as assessed by a medical professional; and Part IV involves the assessment of motor complications and symptom variability by a medical professional on a scale in which scores are assigned as follows: 0, normal; 1, very mild; 2, mild; 3, moderate; and 4, severe. Olfactory function was assessed using the Odor Stick Identification Test for the Japanese (OSIT-J; Daiichi Pharmaceutical Co., Ltd., Tokyo, Japan) or a card-based olfactory identification test (Open Essence; Wako Junyaku) to identify 12 odors (Martinez-Martin et al., 2011). RBD was assessed using the Japanese version of the RBD Screening Questionnaire (RBDSQ-J). The RBDSQ-J is a self-administered questionnaire on the behavioral characteristics of RBD consisting of 13 items, with a cut-off score of six points for the group with possible RBD (pRBD), based on previous studies (Miyamoto et al., 2009; Nomura et al., 2011). Each drug was evaluated using the levodopa equivalent daily dose (LEDD). All patients underwent magnetic resonance imaging to exclude other diseases. This study was approved by the Institutional Review Board of Tokyo Women’s Medical University (approval No. 3316) and the Institutional Review Board of each study site and was conducted in accordance with the “Ethical Guidelines for Clinical Research in Japan” and the “Declaration of Helsinki”. Written informed consent was obtained from all patients before the study began.

### 2.3. Statistical analysis

Results are expressed as mean ± SD. JMP Pro statistical software (version 16; SAS Institute, Tokyo, Japan) was used for statistical analyses. The relationship between the total score and sub-item scores of the MDS-UPDRS Part I and age, disease duration, and MDS-UPDRS Parts II, III, and IV was analyzed using regression analysis. Statistical significance was set at *p* < 0.05 and *r* > 0.20. The patients were divided into two groups: <70 years old (<70 years group), ≥70 years old (≥70 years group), <5 years after the onset of motor symptoms (early group), and > 5 years old (advanced group). The total and sub-item scores of the MDS-UPDRS Part I were compared between the <70 years and ≥ 70 years groups, the early-stage and advanced-stage groups, and males and females using Wilcoxon’s test. Factors (age, sex, disease duration, MDS-UPDRS Part II–IV, RBDSQ-J, and olfactory disorder) contributing to the MDS-UPDRS Part I total score were examined using logistic analysis.

## 3. Results

### 3.1. Patients’ background

This study included 431 patients with PD without dementia (202 males and 229 females), aged 67.7 ± 8.2 years (age, 42–85 years) with a mean duration of disease of 6.4 ± 4.7 years (duration, 0.5–20 years), who visited one of the neurology departments of six hospitals in Japan. The mean MMSE was 28.5 points, the mean LEDD was 463.2 mg/day, and the HY scale (on status) was I for 55 patients, II for 260, III for 95, IV for 16, and V for 5. The mean total scores for MDS-UPDRS Parts I–IV were 9.9 ± 6.3, 12.3 ± 8.5, 26.4 ± 13.3, and 1.8 ± 3.2, respectively. pRBD was found in 32.5% of the patients, and the average number of correct answers on the olfactory identification test was 4.8 (Table 1).

**Table 1:** Clinical characteristics of total patients with Parkinson’s disease.

Number of patients	431
Gender (male/female)	202/229
Age (years)	67.7 ± 8.2 (42–85)
Disease duration (years)	6.4 ± 4.7 (0.5–20)
Mini-mental state examination	28.5 ± 1.6 (24–30)
Hoehn and Yahr stage (on phase)	1: 55, 2: 260, 3: 95, 4: 16, 5: 5
Levodopa-equivalent daily dose (mg/day)	463.2 ± 389.2 (0–1500)
MDS UPDRS Part I total score	9.9 ± 6.3 (1–41)
Part II total score	12.3 ± 8.5 (0–42)
Part III total score	26.4 ± 13.3 (2–69)
Part IV total score	1.8 ± 3.2 (0–21)
RBD SQ-J score Patient numbers of pRBD	4.4 ± 2.9 (0–13) 32.5%
Correct answer of odor identification test (422)	4.8 ± 2.8 (0–12)

Values are mean ± SD (range). MDS-UPDRS, Movement Disorder Society Revision of the Unified Parkinson’s disease rating scale; RBDSQ-J, REM sleep behavior disorder screening questionnaire-Japanese version; pRBD, probable REM sleep behavior disorder.

### 3.2. Association of MDS-UPDRS Part I with age and disease duration

**Figure 1:**
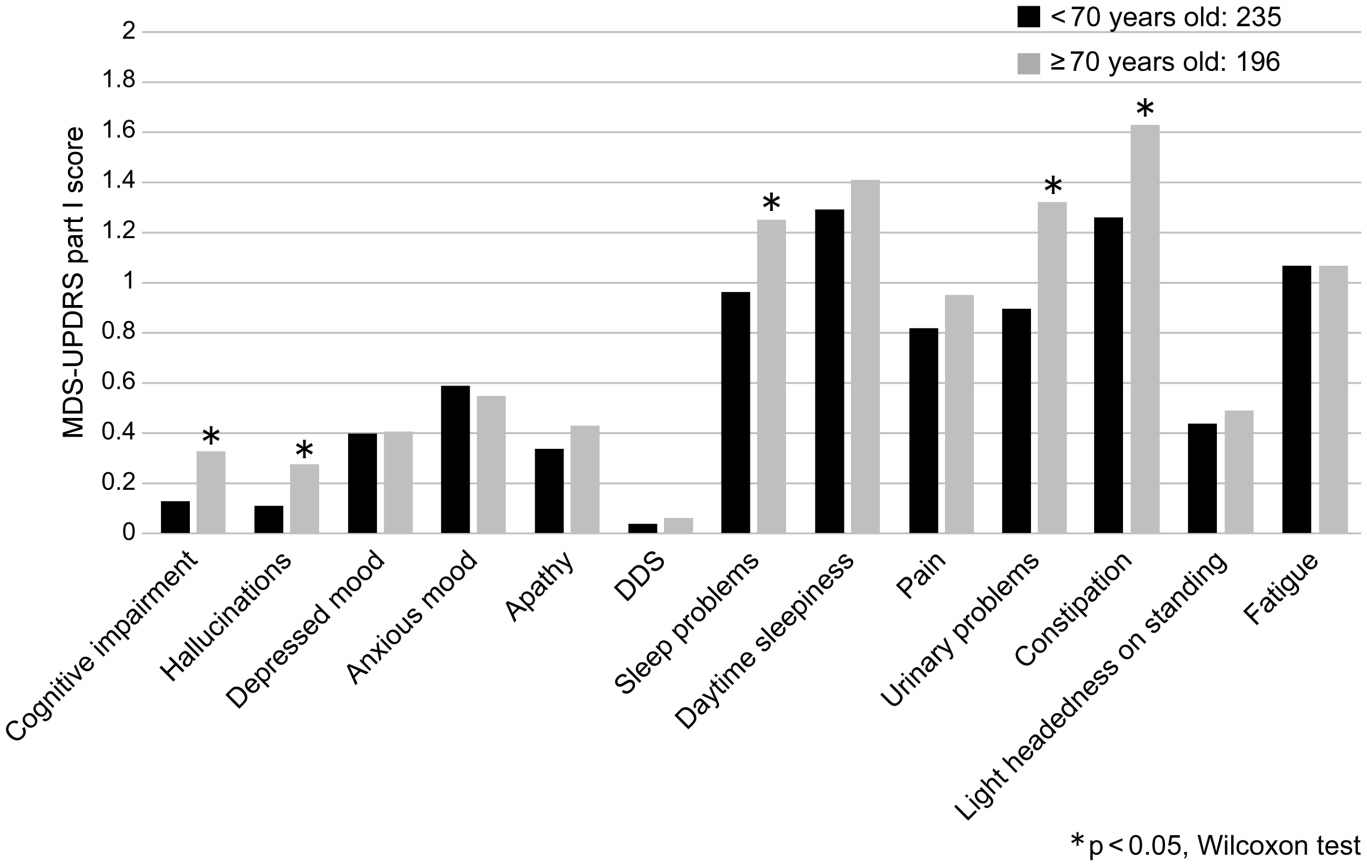
Comparison of MDS-UPDRS Part I sub-item scores and age groups (<70 years and ≥ 70 years). DDS, Dopamine dysregulation syndrome.

The correlation coefficient between the MDS-UPDRS Part I total score and age was low (*r* = 0.190, *p* < 0.0001), evidencing only a correlational trend. More specifically, the MDS-UPDRS Part I sub-items of urinary problems and constipation were found to be weakly correlated with age (Table 2). When the patients were divided into age groups of <70 and ≥70 years, significant differences were found in cognitive impairment, hallucinations, sleep and urinary problems, and constipation (*p* < 0.05; Figure 1).

**Table 2:** MDS-UPDRS Part I in association with age and disease duration.

	Score (Mean ± SD)	Age		Disease duration	
		*r*-value	Value of *p*	*r*-value	Value of *p*
MDS-UPDRS Part I total score	9.9 ± 6.3	0.190	<0.0001	**0.327**	**<0.0001**
Part I items					
Cognitive impairment	0.22 ± 0.51	0.1674	0.0005	0.1172	0.0149
Hallucinations	0.19 ± 0.53	0.1218	0.0114	**0.2900**	**<0.0001**
Depressed mood	0.41 ± 0.71	0.0167	0.7297	0.0825	0.0870
Anxious mood	0.57 ± 0.75	0.0500	0.3013	0.0895	0.0635
Apathy	0.38 ± 0.71	0.0900	0.0629	0.0824	0.0875
Dopamine dysregulation syndrome	0.05 ± 0.30	0.0100	0.8432	**0.2178**	**<0.0001**
Sleep problems	1.09 ± 1.04	0.1425	0.0030	**0.2938**	**<0.0001**
Daytime sleepiness	1.35 ± 0.86	0.0934	0.0527	0.1703	0.0004
Pain	0.88 ± 0.96	0.0716	0.1379	**0.2461**	**<0.0001**
Urinary problems	1.09 ± 1.03	**0.2246**	**<0.0001**	**0.3295**	**<0.0001**
Constipation	1.43 ± 1.20	**0.2513**	**<0.0001**	**0.2141**	**<0.0001**
Light headedness on standing	0.46 ± 0.79	0.3415	0.3709	0.1126	0.0194
Fatigue	1.07 ± 0.96	0.0365	0.4503	0.1500	0.0019

Mean ± SD. Regression analysis.The bold values: *p* < 0.05 and r > 0.20.

**Figure 2:**
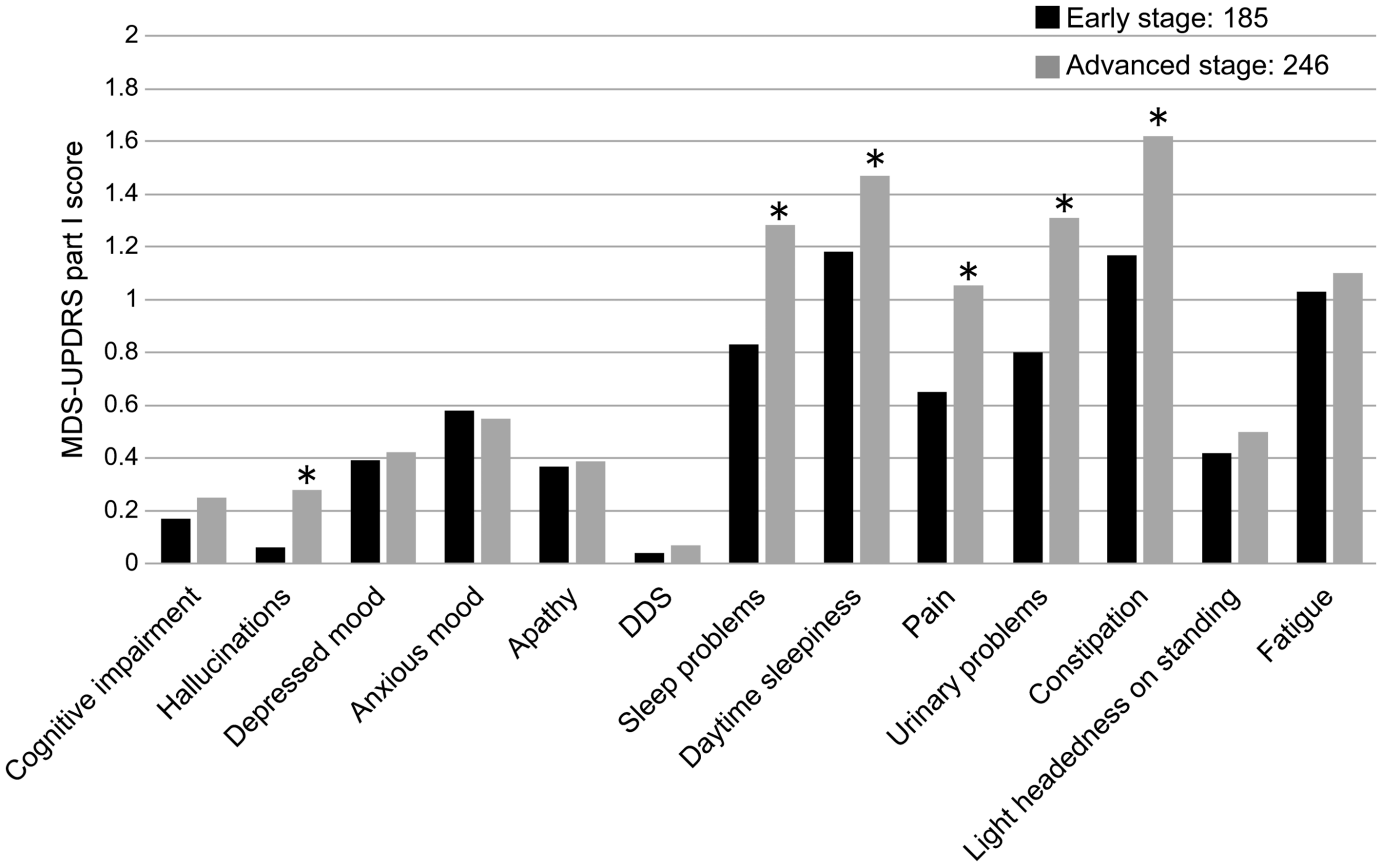
Comparison of MDS-UPDRS Part I sub-item scores and disease duration (early stage, advanced-stage cut-off: 5 years). DDS, Dopamine dysregulation syndrome. *p < 0.05.

The MDS-UPDRS Part I total score and disease duration were positively correlated (*r* = 0.327, *p* < 0.0001), as were the sub-scores of Part I: hallucinations, DDS, sleep and urinary problems, pain, and constipation (Table 2). Comparing the total score of the MDS-UPDRS Part I total score and the early-stage and advanced-stage groups (early stage = < 5 years and advanced stage = ≥ 5 years), no correlation existed with the early-stage PD group (*r* = 0.144, *p* = 0.052); however, a positive correlation was observed with the advanced-stage PD group (*r* = 0.299, *p* < 0.0001). Hallucinations, sleep problems, daytime sleepiness, pain, urinary problems, and constipation were more common among patients with advanced-stage PD (Figure 2).

### 3.3. Sex differences of clinical features in PD patients

**Figure 3:**
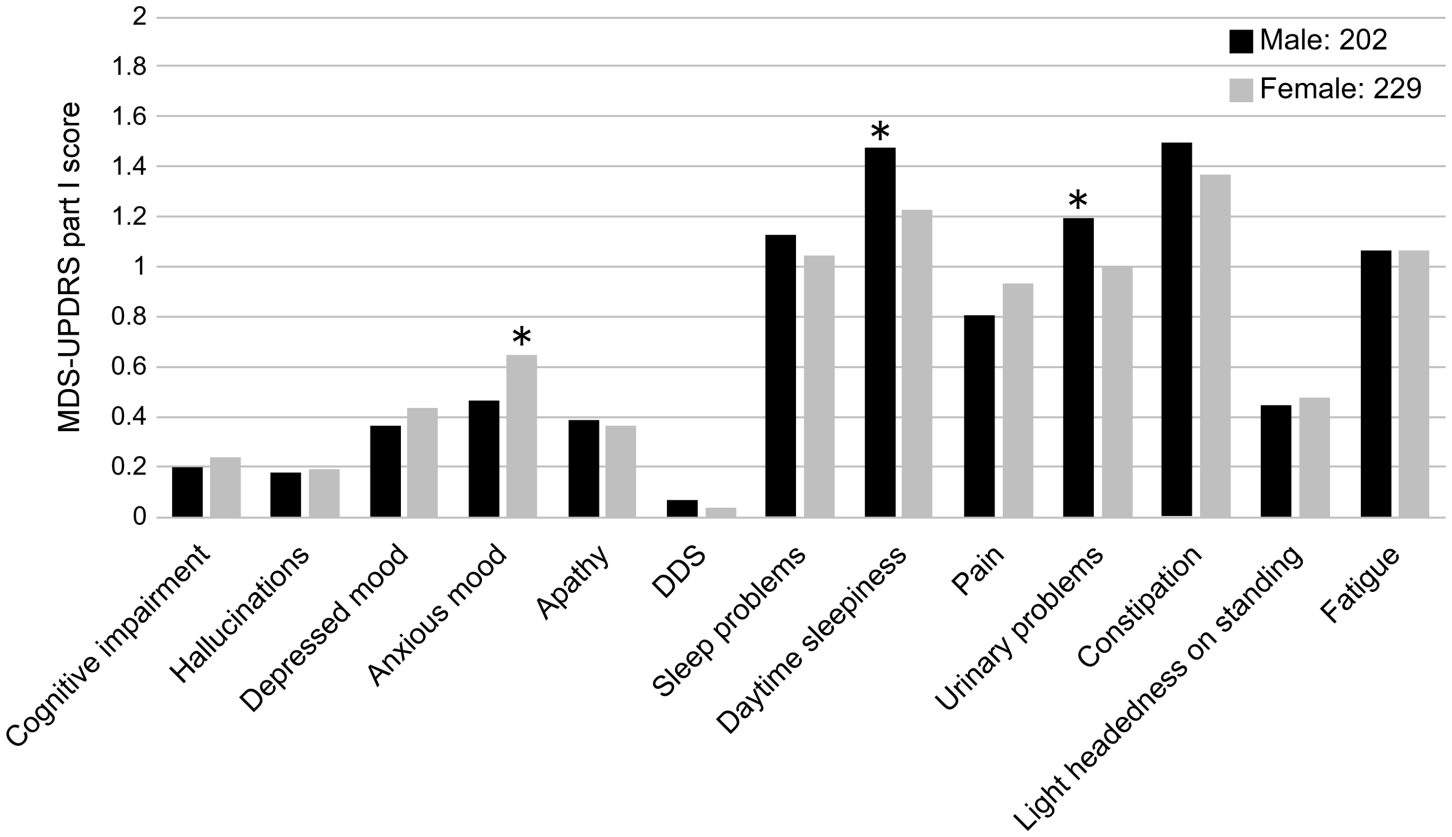
Comparison of MDS-UPDRS Part I sub-item scores and sex (male and female). DDS, Dopamine dysregulation syndrome. *p < 0.05.

The results are summarized in Table 3. A total of 202 male patients and 229 female patients with no significant differences in age, disease duration, or LEDD were included in the two groups. The total scores of the MDS-UPDRS Parts I–IV were more severe in female patients in Part IV only (*p* < 0.05). A comparison of the MDS-UPDRS Part I sub-items revealed significantly higher scores for anxious mood in female patients than male patients (*p* < 0.05) and significantly higher scores for sleep and urinary problems in male patients than female patients (*p* < 0.05; Figure 3). pRBD was more common in male patients, and olfactory identification function was more severe in male patients (*p* < 0.05).

**Table 3:** Sex differences of clinical features in PD patients.

	Male (*n* = 202)	Female (*n* = 229)	Value of *p*
Age (years)	67.0 ± 8.8 (41–84)	68.4 ± 7.6 (39–85)	0.1461
Disease duration (years)	6.4 ± 4.6 (0.5–27)	6.3 ± 4.9 (0.6–27)	0.6241
LED (mg/day)	482.5 ± 386.0 (0–1,200)	446.4 ± 392.1 (0–1,500)	0.2218
MDS-UPDRS Part I total score	10.0 ± 6.0 (0–41)	9.7 ± 6.5 (0–39)	0.2863
MDS-UPDRS Part II total score	12.9 ± 8.5 (0–39)	11.8 ± 8.5 (0–42)	0.1125
MDS-UPDRS Part III total score	27.4 ± 13.8 (0–69)	25.6 ± 12.9 (0–62)	0.2198
**MDS-UPDRS Part IV total score**	**1.6 ± 3.2 (0–21)**	**2.0 ± 3.2 (0–16)**	**0.0393**
**RBD SQ-J score** **Patient numbers of pRBD**	**4.8 ± 3.1 (0–13)** **Yes: 80 (39.6%)**	**4.4 ± 2.9 (0–12)** **Yes: 62 (27.1%)**	**0.0310**
**Correct answer of odor identification test (422)**	**4.2 ± 2.7 (*n* = 200)**	**5.2 ± 2.7 (*n* = 222)**	**0.0002**

Values are mean ± SD (range). Wilcoxon test. The bold values: *p* < 0.05.

### 3.4. Relationship between MDS-UPDRS Part I and Parts II–IV

**Figure 4:**
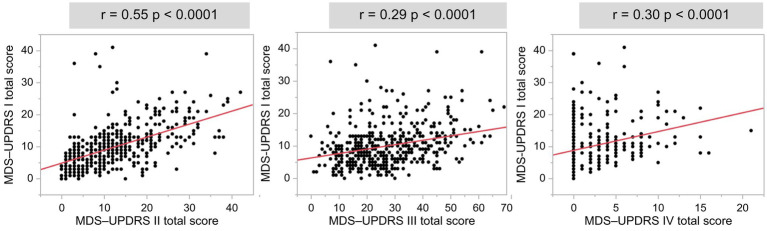
Associations between the MDS-UPDRS Part I total score and Parts II, III, and IV total scores. *r*: *r*-value, *p*: value of *p*.

**Figure 5:**
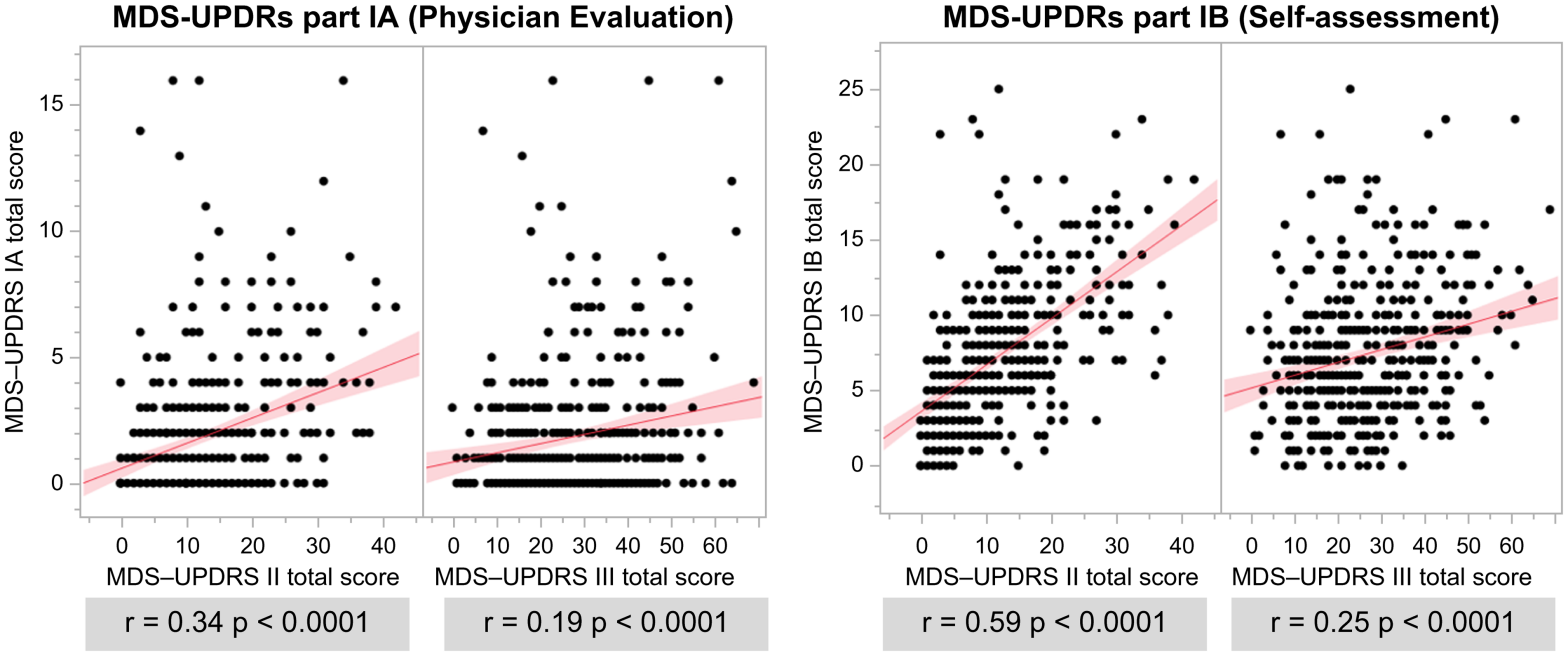
Association of MDS-UPDRS IA and IB with MDS-UPDRS II and III total scores. *r*: *r*-value, *p*: value of *p*.

The total scores of MDS-UPDRS Parts I and II, III, and IV were all significantly positively correlated (Figure 4). Part I of the MDS-UPDRS was divided into Parts IA (physician evaluation) and IB (self-evaluation), and their correlations with Parts II and III were compared. A stronger correlation was observed for Part II than for Part III in the physicians’ evaluation of Part IA. Even in Part IB, which was a self-evaluation, a stronger positive correlation was observed with Part II (Figure 5).

### 3.5. Factors associated with the total score of MDS-UPDRS Part I

An investigation of the MDS-UPDRS Part I total score and patient background (age, sex, and disease duration), MDS-UPDRS Part II–IV total score, RBD (present and absent groups), and the correct number of olfactory identifications showed that the MDS-UPDRS Part I total score was associated with disease duration, Part II total scores, Part IV total scores, and pRBD (Table 4).

**Table 4:** Factors associated with MDS-UPDRS Part I total score.

	Estimated value	Standard error of the mean(SEM)	Value of *p*
Age (years)	0.0393	0.0326	0.2577
Sex (male/female)	−0.1808	0.2615	0.4897
**Disease duration (years)**	**0.1418**	**0.0632**	**0.0253**
**MDS-UPDRS Part II total score**	**0.3169**	**0.0380**	**<0.0001**
MDS-UPDRS Part III total score	−0.0024	0.0228	0.9176
**MDS-UPDRS Part IV total score**	**0.1880**	**0.0922**	**0.0421**
**pRBD (with or without pRBD, RBD SQ-J scores)**	**−0.7154**	**0.2773**	**0.0102**
Correct answer of odor identification test	−0.1741	0.0961	0.0708

Method of least square. The bold values: *p* < 0.05.

## 4. Discussion

### 4.1. Relationship between NMS, age, and disease duration

An FY2020 patient survey by the Ministry of Health, Labor, and Welfare showed that the total number of patients with PD in Japan was 289,000, of whom 234,000 were at least 70 years old, accounting for 81% (FY2020 patient survey: total number of patients, sex/age group × minor disease classification, Ministry of Health, Labor, and Welfare). The definition of elderly PD (i.e., elderly cut-off value) in this study was set at ≥70 years to better reflect clinical practice. Significant differences were observed between the <70-year group and ≥ 70-year group in terms of cognitive impairment, hallucinations, sleep problems, urinary problems, and constipation. This cross-sectional study included older adult patients with late-onset PD and those who grew older with continued treatment.

The pathological features of PD include the loss of midbrain substantia nigra neurons and the presence of eosinophilic inclusions called Lewy bodies, which are formed by the aggregation and deposition of α-synuclein in damaged neurons (Braak et al., 2003). Pathological progression is thought to begin in the dorsal vagus nucleus and olfactory bulb, which then progresses to the locus coeruleus pontine and midbrain substantia nigra, ascends the brain stem, and spreads to the cerebral cortex, with most cases exhibiting Lewy pathology from the peripheral to the central system (Braak et al., 2003). The diversity of NMS in patients with PD may be due to the extensive deposition of synucleins in the central, peripheral, and autonomic nervous systems (Braak et al., 2003; Jellinger, 2011; Schapira et al., 2017).

Regarding the pathological progression of PD, cognitive dysfunction is present in advanced PD (Savica et al., 2010; Kalia and Lang, 2015), but cognitive dysfunction in late-onset PD is already severe at the time of diagnosis (Pagano et al., 2016), and 21 autopsy cases of PD with an onset of ≥80 years developed dementia earlier than that reported in young-onset cases (Rajput and Rajput, 2017). A Japanese study indicated that the prevalence of mild cognitive dysfunction was 17.0% and often in those aged ≥65 years (Ninomiya et al., 2020). Moreover, cognitive dysfunction in PD occurs in approximately 83% of cases and may occur late in the pathological progression of PD or as a result of aging (Hely et al., 2008). Dysuria in PD patients is characterized by an overactive bladder, with nocturia being the most common at ≥60% and urinary incontinence at 26% for males and 28.5% for females (Sakakibara et al., 2012). Age-related vascular endothelial dysfunction, autonomic neuropathy, and inflammation are also important causes of an overactive bladder, with the number of patients and frequency increasing with age, which are major factors in the impairment of QOL among older adults (Homma et al., 2006; Irwin et al., 2006). Constipation in PD is often observed prior to PD diagnosis since α-synuclein aggregates have been confirmed in the enteric plexus (Wakabayashi et al., 1989). Constipation in the older adults is caused by a longer colon transit time and a higher rectal sensory threshold than in young people, and the 2019 Comprehensive Survey of Living Conditions by the Ministry of Health, Labor, and Welfare showed that 72.3% of females aged ≥65 years felt constipated, as did 64.1% of males aged ≥65 years. Therefore, cognitive dysfunction, dysuria, and constipation are important not only for the pathological progression of PD but also for older adult individuals.

Hallucinations often occur in the advanced stages of PD, particularly in the last 5 years, owing to pathological changes associated with the degeneration of the central nervous system, particularly the acetylcholine system, and the appearance of Lewy bodies (Kempster et al., 2010). The risk factors for hallucinations include old age, severe motor symptoms of PD, cognitive dysfunction, visual impairment, and sleep disturbances (Diederich et al., 2009). Regarding the pharmacokinetics of L-dopa in patients with elderly onset PD, higher doses of L-dopa are required in older adult patients than other patients with PD because of lower peak blood levels and increased time to reach peak blood levels (Nagayama et al., 2011), which may also be associated with an increased risk of hallucinations (Kalia and Lang, 2015). These findings suggest that hallucinations are often observed in older adult patients with PD undergoing long-term treatment. A 2017 report found that 96.5% of patients with PD exhibited sleep disorders such as RBD, sleep apnea, and excessive daytime drowsiness (Arnulf, 2005; Sobreira-Neto et al., 2017). PD-derived disorders of the sleep–wake center, nocturnal motor symptoms, cognitive dysfunction, and neurological symptoms, such as hallucinations, drugs, and nocturia, are complications of sleep disturbances. The risk factors for daytime drowsiness include disease duration, severity of motor symptoms, and total PD medication dose (Arnulf, 2005; Kang et al., 2017). Therefore, hallucinations, sleep problems, and daytime sleepiness may be related to disease duration and drug use, in addition to an older adult background.

There was a positive correlation between the MDS-UPDRS Part I total score and disease duration, whereas the advanced-period group showed a correlation with the Part I total score, although the early-stage group did not. The response to dopamine replacement therapy is good in the 3–5 year period after PD diagnosis, after which motor complications such as the wearing-off phenomenon and dyskinesia appear as side effects of L-dopa and the treatment response becomes poor (Aquino and Fox, 2015). A report on patients with early-onset PD (within 5 years) indicated that NMS progresses in parallel with motor symptoms (Santos-Garcia et al., 2020). In this study, the fact that no correlation was observed between NMS and the early-stage group may be because the neurodegenerative area was not extensive and patients with early-stage PD responded well to dopamine replacement therapy and had mild progression of NMS.

Comparisons between the early- and advanced-stage groups showed significant differences in hallucinations, sleep problems, daytime sleepiness, pain, urinary problems, and constipation compared with the MDS-UPDRS Part I sub-items. In PD cases, α-synuclein aggregates appear in the enteric plexus, and constipation is often seen prior to PD diagnosis (Wakabayashi et al., 1989). With pathological progression, hallucinations are associated with the occipital lobe; sleep disturbance is associated with the hypothalamus and reticular formation; pain is associated with the basal ganglia, coeruleus, raphe nucleus, amygdala, and thalamus; and dysuria, particularly urination function, is associated with the hypothalamus, cerebrum, frontal cortex, and basal ganglia, making the disease highly severe (Schapira et al., 2017). Drowsiness can be exacerbated by dopamine receptor agonists (Lim and Lang, 2010), and a multivariate analysis of patients with advanced PD showed that LEDD ≥400 mg/day was associated with an increased MDS-UPDRS Part I total score (Maeda et al., 2017).

### 4.2. Relationship between NMS and sex

Male patients had a higher severity of pRBD, daytime sleepiness, urinary problems, and olfactory disturbances than female patients. RBD is more common in male patients (Cerri et al., 2019; Iijima et al., 2021), and olfactory disturbances are more common in male patients (Iijima et al., 2011, 2021); these results are in agreement with those of previous studies. Excessive daytime drowsiness was found in 20–50% of patients with PD, and risk factors included male sex, disease duration, severity of motor symptoms, and drugs (Kang et al., 2017). Drowsiness is presumed to be caused by PD-derived disorders of the sleep–wake system, RBD, and drugs. The basal ganglia have an inhibitory effect on urination; therefore, PD patients with basal ganglia lesions have an overactive bladder (Sakakibara et al., 2008). When considering the age at the onset of PD, male patients are more likely to develop obstructive dysuria, which coincides with the age of prostatic hyperplasia (Homma et al., 2006), and middle-aged and older adult females are prone to stress urinary incontinence (Sakakibara et al., 2008). The frequency of dysuria, including urinary incontinence, in PD cases has been reported to be 53% in males and 63% in females (Sakakibara et al., 2001). The patients in the present study were older than those in previous reports; therefore, it is possible that dysuria was significantly more severe in males than in females.

Female patients had higher MDS-UPDRS Part IV total scores and anxious moods than male patients. In the West, PD onset is more common in male patients than in female patients, and the incidence of PD in males and postmenopausal female patients is roughly equivalent. Therefore, it is presumed that female hormones may prevent the risk of PD onset and have neuroprotective effects (Cerri et al., 2019). There are also sex differences in the bioavailability of L-dopa, suggesting the involvement of estrogen (Kumagai et al., 2014). Anxiety and dyskinesia are common among women in Western countries (Cerri et al., 2019). In Japan, the prevalence rate is 0.56 in females and the incidence rate is 0.61 in females, with both being higher in females (Yamawaki et al., 2009) than in males. The present study also showed significant differences in anxiety and higher rates in females than in males, which is consistent with previous reports. A report on the pharmacokinetics of L-dopa in older adult patients involved a body-weight-adjusted comparison between males and females, and the results showed that the L-dopa area under the blood concentration-time curve (AUC) was significantly higher in females (Kumagai et al., 2014) than in males. In a study involving 220 patients with PD in Japan, age and female sex were associated with an AUC of 4 h (Nishikawa et al., 2020). Female patients had a significantly greater bioavailability of L-dopa than male patients, and it was thought that female patients had more motor complications during the drug treatment of PD than that in male patients. The present study did not distinguish between MDS-UPDRS Part IV total score dyskinesia and the wearing-off phenomenon, and further examination is necessary. These results indicate that the clinical symptoms of NMS in PD differ between males and females and that it is necessary for clinicians to pay attention when treating patients with PD.

### 4.3. Relationship between NMS and each part of MDS-UPDRS

A positive correlation was observed between MDS-UPDRS Part I and Part II/III total scores, suggesting that NMS in PD progresses in parallel with motor symptoms. Part I of the MDS-UPDRS was divided into Parts IA (physician evaluation) and IB (self-evaluation), which were compared with Parts II (self-evaluation of motor symptoms) and III (physician evaluation of motor symptoms). A 2022 report that examined patients with PD with an HY scale of 1 or 2 showed a positive correlation between MDS-UPDRS Parts IA/IB and II, no correlation between Parts IA and III, and a correlation between parts IB and III (Zolfaghari et al., 2022). The findings of this study including patients with PD with an HY scale of 1–5 were consistent with those of previous reports. The evaluation of side effects over time by physicians and patients showed that physician evaluations had lower values than patient evaluations at all time points (Basch, 2010). A previous study indicated that healthcare professionals underestimated adverse events in patients (Sonn et al., 2013). There was also a discrepancy between physician and patient evaluations in the present study, and NMS was more correlated with the Part II total score, which is a subjective symptom questionnaire, suggesting that subjectivity is correlated.

The MDS-UPDRS Part I and Part IV total scores were positively correlated. Patients in the advanced stages of PD may experience the wearing-off phenomenon, dyskinesia, and PD patients may face many symptoms associated with NMS (Sato et al., 2006).

### 4.4. Factors that contribute most to NMS

The factors that contributed to the MDS-UPDRS Part I total score were disease duration, MDS-UPDRS Part II total score, Part IV total score, and pRBD, with the Part II total score being the largest contributing factor. Neurodegenerative areas become extensive due to pathological progression; therefore, NMS is thought to become more severe during the disease duration (Braak et al., 2003; Jellinger, 2011; Schapira et al., 2017). The MDS-UPDRS Part IV total score, which evaluates motor complications, also evaluates dyskinesia and the wearing-off phenomenon that is observed in the advanced stages of PD, and NMS is thought to become severe because of the high score during the advanced stages of PD (Sato et al., 2006; Aquino and Fox, 2015). It has been reported that pRBD contributes to the MDS-UPDRS Part I total score (Iijima et al., 2021), and this is similar to the results of the present study. Moreover, NMS by self-question evaluation contributed the most to decreased motor dysfunction. Medical staff should avoid relying solely on their own evaluation and value the subjective symptoms of patients.

### 4.5. Limitations

This study has some limitations. First, cognitive impairment may have been underestimated because patients with obvious cognitive decline were excluded. Second, the motor score (MDS-UPDRS Part III) was evaluated in the “on” state, and this cannot be compared with the “off” state, and further study is needed in the future. Furthermore, this study was a cross-sectional study within a single ethnic group and could have been influenced by potential biases introduced through the use of different assessment tools with respect to olfactory function. We believe that future prospective studies involving patients from various regions and cultures must be conducted.

## 5. Conclusion

NMS, assessed using a self-administered questionnaire, was highly severe in older patients, those with a longer disease duration and subjective and objective motor function decline. There were also differences between the sexes. Additionally, the background and NMS of each patient must be evaluated to provide individualized medical care that satisfies different needs and improves the QOL of patients with PD.

## Data availability statement

The raw data supporting the conclusions of this article will be made available by the authors, without undue reservation.

## Ethics statement

The studies involving humans were approved by the Institutional Review Board of Tokyo Women’s Medical University (approval No. 3316) and the Institutional Review Board of each study site and was conducted in accordance with the “Ethical Guidelines for Clinical Research in Japan” and the “Declaration of Helsinki.” The studies were conducted in accordance with the local legislation and institutional requirements. The participants provided their written informed consent to participate in this study.

## Author contributions

RM, MI, YO, KS, FY, SN, TO, and KK: conceptualization. RM, MI, YO, KS, FY, SN, and TO: data curation. RM and MI: formal analysis and methodology. MI, YO, KS, FY, SN, and TO: investigation. KK: project administration and supervision. RM: writing – original draft. YO, KS, FY, SN, TO, and KK: writing – review and editing. All authors contributed to the article and approved the submitted version.

## Conflict of interest

The authors declare that the research was conducted in the absence of any commercial or financial relationships that could be construed as a potential conflict of interest.

## Publisher’s note

All claims expressed in this article are solely those of the authors and do not necessarily represent those of their affiliated organizations, or those of the publisher, the editors and the reviewers. Any product that may be evaluated in this article, or claim that may be made by its manufacturer, is not guaranteed or endorsed by the publisher.
